# Supplementary material for: Strength and durability of indirect protection against SARS-CoV-2 infection through vaccine and infection-acquired immunity
Source: Nat Commun. 2025 Jan 29;16:1090. doi: 10.1038/s41467-024-55029-9 (PMC11779853; doi:10.1038/s41467-024-55029-9)
Supplement: Supplementary file 2 — Reporting Summary [file 41467_2024_55029_MOESM2_ESM.pdf]

nature portfolio

Corresponding author(s): Nathan Lo

Last updated by author(s): Nov 25, 2024

Reporting Summary

Nature Portfolio wishes to improve the reproducibility of the work that we publish. This form provides structure for consistency and transparency in reporting. For further information on Nature Portfolio policies, see our Editorial Policies and the Editorial Policy Checklist.

Statistics

For all statistical analyses, confirm that the following items are present in the figure legend, table legend, main text, or Methods section.

n/a	Confirmed	
☐	☒	The exact sample size (*n*) for each experimental group/condition, given as a discrete number and unit of measurement
☐	☒	A statement on whether measurements were taken from distinct samples or whether the same sample was measured repeatedly
☐	☒	The statistical test(s) used AND whether they are one- or two-sided<br>*Only common tests should be described solely by name; describe more complex techniques in the Methods section.*
☐	☒	A description of all covariates tested
☐	☒	A description of any assumptions or corrections, such as tests of normality and adjustment for multiple comparisons
☐	☒	A full description of the statistical parameters including central tendency (e.g. means) or other basic estimates (e.g. regression coefficient) AND variation (e.g. standard deviation) or associated estimates of uncertainty (e.g. confidence intervals)
☐	☒	For null hypothesis testing, the test statistic (e.g. *F*, *t*, *r*) with confidence intervals, effect sizes, degrees of freedom and *P* value noted<br>*Give P values as exact values whenever suitable.*
☒	☐	For Bayesian analysis, information on the choice of priors and Markov chain Monte Carlo settings
☒	☐	For hierarchical and complex designs, identification of the appropriate level for tests and full reporting of outcomes
☒	☐	Estimates of effect sizes (e.g. Cohen's *d*, Pearson's *r*), indicating how they were calculated

Our web collection on statistics for biologists contains articles on many of the points above.

Software and code

Policy information about availability of computer code

Data collection	Data was collected as part of public health SARS-CoV-2 surveillance program in California state prisons.
Data analysis	Code is publicly available via Github: http://github.com/sophttan/covid-indirects (stable doi in References). Analysis was conducted with R statistical software (version 4.3.1).

For manuscripts utilizing custom algorithms or software that are central to the research but not yet described in published literature, software must be made available to editors and reviewers. We strongly encourage code deposition in a community repository (e.g. GitHub). See the Nature Portfolio guidelines for submitting code & software for further information.

Data

Policy information about availability of data

All manuscripts must include a data availability statement. This statement should provide the following information, where applicable:

- Accession codes, unique identifiers, or web links for publicly available datasets
- A description of any restrictions on data availability
- For clinical datasets or third party data, please ensure that the statement adheres to our policy

Data requests may be made to California Correctional Health Care Services and are subject to controlled access.

Research involving human participants, their data, or biological material

Policy information about studies with human participants or human data. See also policy information about sex, gender (identity/presentation), and sexual orientation and race, ethnicity and racism.

Reporting on sex and gender	Sex is reported in Table 1. 97% of the study population self-reported as male.
Reporting on race, ethnicity, or other socially relevant groupings	Race of the study population is reported in Table 1. Race was a self-reported demographic measure.
Population characteristics	Additional population characteristics related to COVID-19 are included in Table 1, Figure 1, and the Results. We report on COVID-19 vaccine and prior SARS-CoV-2 infection status of the study population.
Recruitment	Study population were persons incarcerated in the California state prison system as part of surveillance testing for SARS-CoV-2. Participants did not receive any compensation.
Ethics oversight	This study was approved by the IRB at Stanford University and UCSF. The IRB included a waiver of consent given use of retrospective secondary data without direct identifiers that were collected for public health surveillance and deemed to be minimal risk (see Supplementary Notes).

Note that full information on the approval of the study protocol must also be provided in the manuscript.

Field-specific reporting

Please select the one below that is the best fit for your research. If you are not sure, read the appropriate sections before making your selection.

☒ Life sciences ☐ Behavioural & social sciences ☐ Ecological, evolutionary & environmental sciences

For a reference copy of the document with all sections, see nature.com/documents/nr-reporting-summary-flat.pdf

Life sciences study design

All studies must disclose on these points even when the disclosure is negative.

Sample size	The final sample size included 9,625 residents with testing data that met study criteria. Our study included 4,640 cases and 7,824 controls. Sample sizes were not predetermined. We evaluated the power of the final study sample size after applying strict study inclusion criteria.
Data exclusions	Residents were excluded if they were not incarcerated over the entire pandemic to ensure more complete reporting of prior infection and testing history. Residents were excluded if they were missing housing data. Cases and controls were excluded if had a recent infection or did not meet additional housing criteria (must stay in 2-person rooms). Study inclusion and exclusion criteria were pre-specified (see Supplementary Information and pre-analysis plan) and are outlined in the Methods section (COVID-19 cases/controls) and Figure 2.
Replication	Analysis was conducted with automated code scripts, no further replication studies were conducted.
Randomization	None, this was an observational study.
Blinding	Person-level data was de-identified. There was no randomization in this study, no further blinding was done.

Reporting for specific materials, systems and methods

We require information from authors about some types of materials, experimental systems and methods used in many studies. Here, indicate whether each material, system or method listed is relevant to your study. If you are not sure if a list item applies to your research, read the appropriate section before selecting a response.

Materials & experimental systems

n/a	Involved in the study	
☒	☐	Antibodies
☒	☐	Eukaryotic cell lines
☒	☐	Palaeontology and archaeology
☒	☐	Animals and other organisms
☐	☒	Clinical data
☒	☐	Dual use research of concern
☒	☐	Plants

Methods

n/a	Involved in the study	
☒	☐	ChIP-seq
☒	☐	Flow cytometry
☒	☐	MRI-based neuroimaging

Clinical data

Policy information about clinical studies

All manuscripts should comply with the ICMJE guidelines for publication of clinical research and a completed CONSORT checklist must be included with all submissions.

Clinical trial registration	N/A; This was a retrospective review of anonymized secondary data. There was no prospective data collection or experimentation.
Study protocol	The analytic protocol is described in the Methods/Appendix. A pre-analysis plan is available here: https://github.com/sophttan/covid-indirects, along with the full analytic code (stable doi in References).
Data collection	We conducted a retrospective study using anonymized data from the California Correctional Health Care Services (CCHCS) and their system-wide SARS-CoV-2 surveillance program of 177,319 residents across 35 California state prisons to measure vaccine-derived and infection-acquired indirect protection. The study period was from December 15, 2021, to December 15, 2022, to study Omicron variants/subvariants.
Outcomes	The model outcome was SARS-CoV-2 infection (case or control), in order to estimate indirect protection as defined in the Methods.

Plants

Seed stocks	N/A
Novel plant genotypes	N/A
Authentication	N/A
